# Simulation of stent deployment in a realistic human coronary artery

**DOI:** 10.1186/1475-925X-7-23

**Published:** 2008-08-06

**Authors:** Frank JH Gijsen, Francesco Migliavacca, Silvia Schievano, Laura Socci, Lorenza Petrini, Attila Thury, Jolanda J Wentzel, Anton FW van der Steen, Patrick WS Serruys, Gabriele Dubini

**Affiliations:** 1 Department of Biomedical Engineering, Thoraxcentre Ee2322, Erasmus Medical Center Rotterdam, P.O. Box 2040, 3000 CA, Rotterdam, The Netherlands; 2 Laboratory of Biological Structure Mechanics, Structural Engineering Department, Politecnico di Milano, Milan, Italy; 3 Cardiothoracic Unit, University College of London Institute of Child Health and Great Ormond Street Hospital for Children, London, UK

## Abstract

**Background:**

The process of restenosis after a stenting procedure is related to local biomechanical environment. Arterial wall stresses caused by the interaction of the stent with the vascular wall and possibly stress induced stent strut fracture are two important parameters. The knowledge of these parameters after stent deployment in a patient derived 3D reconstruction of a diseased coronary artery might give insights in the understanding of the process of restenosis.

**Methods:**

3D reconstruction of a mildly stenosed coronary artery was carried out based on a combination of biplane angiography and intravascular ultrasound. Finite element method computations were performed to simulate the deployment of a stent inside the reconstructed coronary artery model at inflation pressure of 1.0 MPa. Strut thickness of the stent was varied to investigate stresses in the stent and the vessel wall.

**Results:**

Deformed configurations, pressure-lumen area relationship and stress distribution in the arterial wall and stent struts were studied. The simulations show how the stent pushes the arterial wall towards the outside allowing the expansion of the occluded artery. Higher stresses in the arterial wall are present behind the stent struts and in regions where the arterial wall was thin. Values of 200 MPa for the peak stresses in the stent strut were detected near the connecting parts between the stent struts, and they were only just below the fatigue stress. Decreasing strut thickness might reduce arterial damage without increasing stresses in the struts significantly.

**Conclusion:**

The method presented in this paper can be used to predict stresses in the stent struts and the vessel wall, and thus evaluate whether a specific stent design is optimal for a specific patient.

## Background

Stent implantation is nowadays a common interventional procedure with a high rate of success if compared to angioplasty [1]. A vascular stent is a small slotted metal tube, which is inserted into an artery at the site of narrowing to act as an internal scaffolding or support to the blood vessel. A process called in-stent restenosis limits the clinical success of bare metal stents. In-stent restenosis is caused by neointimal hyperplasia and the process consists of various phases, including an inflammatory phase, a granulation or cellular proliferation phase, and a phase of remodelling involving extracellular matrix protein synthesis [2].

Neointimal hyperplasia is initiated by arterial injury sustained during percutaneous coronary intervention. The arterial injury, in terms of medial disruption, intimal denudation and the presence of stent struts, trigger a cascade of processes. These include production of growth factors and cytokines, which trigger proliferation and migration of smooth muscle cell and allow production of extracellular matrix. Together these processes significantly compromise intra-arterial lumen leading to restenosis.

Despite the recent advent of drug-eluting stents, which lead to a reduction of neointima hyperplasia, there are still important risk factors associated with restenosis [3].

It is well known that neotimimal hyperplasia is influenced by local variations in biomechanical environment, including local arterial wall stress [4] and strain distribution and blood flow induced wall shear stress [5]. The outward force of the stent against the vessel wall creates non-physiologic stresses and strains. The stent design as well as the modality of stent expansion could produce a different arterial response to the mechanical action induced by the stent. Knowledge of the local stress distribution in the vessel wall, generated during the intervention, may help in understanding some aspects of neointimal hyperplasia. Furthermore, there is a trend to design stents with thinner stent struts to reduce arterial injury [6]. Reduction of stent strut thickness might lead to higher stresses in the struts, possibly leading to stent strut fracture. This in turn will increase arterial injury dramatically, thus promoting neointimal hyperplasia.

Recent studies of angioplasty and stenting procedures by means of computational structural analyses [7-22] aim at predicting and calculating the stress state generated after a percutanous intervention. These research endeavours in the stenting arena illustrate that there is a great interest to study the role of biomechanical factors on the development of restenosis after stenting or/and angioplasty. However, apart from the work by Holzapfel et al. [10,14] and Kiousis et al. [22], none of the above mentioned studies included a patient derived arterial geometry to simulate stent deployment.

In this work we study the deployment of a stent in a patient derived 3D reconstruction of a mildly stenosed coronary artery by means of the finite element method. We will investigate the effect of stent deployment on stresses and strains in the vessel wall and relate these parameters to neointimal hyperplasia in patients. Furthermore, stresses in the stent struts are computed to investigate stent strut fracture. The influence of strut thickness on both the stress in the vessel wall and in the stent itself will be studied. In the future, this method could be applied prior to a stent procedure in order to choose which is the best device to be implanted and to enable an interventional cardiologist to investigate the effects of different procedures on stresses and displacements in the arterial wall.

## Methods

**Figure 1:**
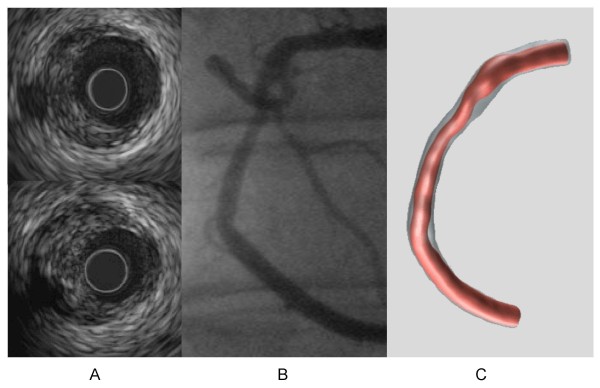
**The intravascular ultrasound images of a mildly stenosed right coronary artery (panel A), the corresponding contrast angiogram (panel B) and the 3D reconstruction (panel C)**.

A 57-year old male presented with unstable angina and underwent coronary angiography, on which a borderline stenotic right coronary artery (RCA) lesion (Figure 1a) was detected. This lesion was successfully treated with a Bx Velocity stent (Cordis, Johnson and Johnson, Warren, NJ, USA). Biplane angiography and intravascular ultrasound (IVUS) in the RCA were applied prior to the intervention and these measurements were combined to obtain the 3-dimensional shape of the lumen and the vessel wall (Figure 1c). These data served as an input to generate a finite element model of the wall of the pre-interventional RCA.

We also generated a finite element model of the unexpanded stent. Subsequently, the unexpanded stent model was introduced in the pre-interventional model of the RCA and virtually deployed in order to mimic the actual in vivo stenting procedure. A finite element program was used to compute the deployment of the stent by means of increasing inflation pressure.

### 3 D reconstruction technique

The 3D reconstruction technique for the coronary arteries is a combination of angiography and IVUS [23]. Briefly, a sheath-based IVUS catheter (CVIS 2.9F, Sunnyvale, CA, USA) is positioned in the vessel segment and is filmed with a biplane angiographic system (Siemens, Bicor, Erlangen, Germany). To eliminate respiratory and cardiac motion artifacts, a single biplane view at end-diastole of the catheter position is selected and digitized. From the biplane views, the transducer path is reconstructed in 3D space [24]. In addition, IVUS images are collected at end-diastole using an ECG triggered, motorized pull back system operating with a step size of 0.5 mm (TomTec, Munich, Germany). Subsequently, the IVUS frames are digitized and analyzed with a semi-automatic contour detection program [25]. Output of the program consists of lumen contours, signifying the lumen/wall interface and media contours signifying the media/adventitia interface.

Subsequently, the lumen contours are filtered and positioned perpendicular onto the reconstructed 3D catheter path, which serves as a backbone for the reconstruction. The angular position of the ultrasound transducer, and thus of the ultrasound images, is determined from a comparison between simulated silhouette images derived from the initial 3D reconstruction with the actual coronary angiogram as shown in Figures 1b and 1c[23].

Finally, a 3D reconstruction of lumen and vessel wall is generated with the software package Rhinoceros 2.0 Evaluation CAD program (McNeel & Associates, Indianapolis, IN, USA) utilizing B-splines.

### Finite element models

**Figure 2:**
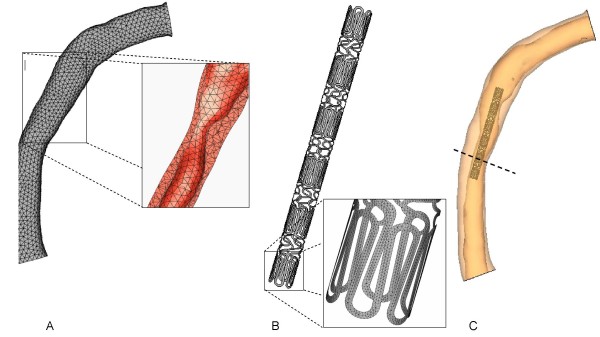
**Mesh of the vessel with an inset showing the internal lumen (panel A)**. Geometry and mesh of the stent in its undeformed configuration (Panel B). The model of the right coronary artery with the unexpanded stent is show in panel C. The location of the minimal lumen diameter (MLD) is indicated by the dashed line.

Once the surfaces were created the volume between them was generated and by means of the software GAMBIT (ANSYS, Inc., Canonsburg, PA USA) a finite element mesh was built. To simplify the computational analysis the distal part of the coronary without any stenotic tract was removed. The model (Figure 2a) was discretized by means of 10845 10-node modified tetrahedron, hybrid with linear pressure with a corresponding number of nodes of 20842. The hybrid formulation was chosen in order to satisfy the incompressibility constraint of the material. To describe the mechanical behaviour of the artery, a hyperelastic isotropic constitutive model along with hybrid modified elements is adopted. In particular, the constitutive law is based on the following strain energy density function U:

*U *= 0.04·(*I*_1 _- 3) + 0.003·(*I*_2 _- 3)^2 ^+ 0.085·(*I*_2 _- 3)^3^

where *I*_1 _and *I*_2 _are the first and the second invariants of the Cauchy-Green tensor [26], The stress-elongation curve obtained recalls the typical behaviour of arteries submitted to tensile tests [27,28] obtained from biological samples. The values adopted produced a slightly stiffer artery if compared with a previous study of ours [11].

The geometry of Bx Velocity stent has an inner diameter in the unexpanded configuration of 0.9 mm and a thickness of the stent is 0.1 mm (Figure 2b). The length of the stent is 13 mm. The stent is discretized by means of 3-node shell elements for a total number of 21066. The stent is made of AISI 316L stainless steel. The inelastic constitutive response is described through a Von Mises-Hill plasticity model with hardening. The Young modulus is 196 GPa, the Poisson ratio 0.3, the yield stress 205 MPa [29]. Using a kinematic hardening the yield stress was reduced to 105 MPa to take into account the crimping [30].

The influence of strut thickness is evaluated with an additional simulation where the value of the thickness is increased to 0.14 mm.

### Finite element solution procedure

The stent was positioned inside the coronary artery (Figure 2c) and expanded under load control conditions untill a pressure of 1.0 MPa was reached. The artery was constrained in the proximal and distal sections preventing any displacements and rotations. The stent was constrained to allow only radial displacements. In particular, three nodes in the central section of the stent were constrained in the axial direction to avoid any migration of the stent inside the vessel. Furthermore, nodes belonging to the stent surface were constrained in the tangential direction to avoid any rotation of the stent inside the vessel.

A large deformation analysis was performed using ABAQUS/Standard commercial code (Abaqus Inc., Providence RI, USA). The nonlinear problem, due to material plasticity and contact constraint, was solved using a Newton-Raphson's method. To model possible interactions between specific model portions, frictionless contact surfaces were introduced.

## Results

The results are presented in terms of computed deformation patterns, stress distribution in the arterial wall and in the stent struts.

**Figure 3:**
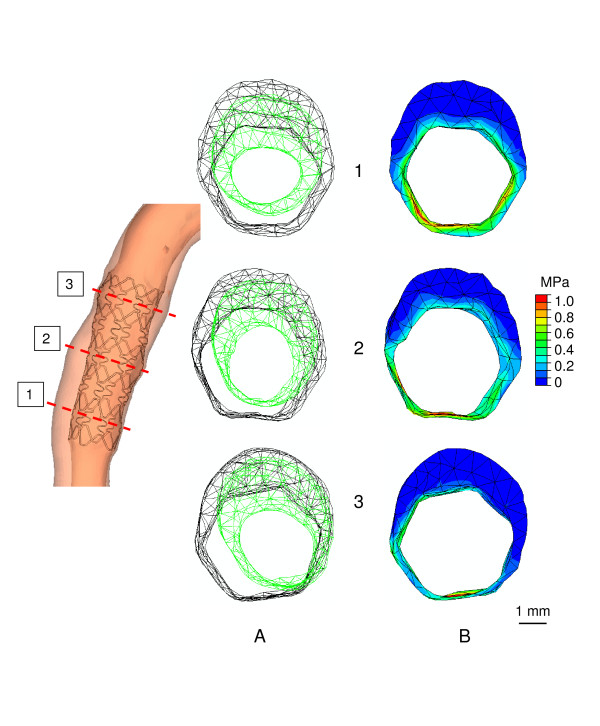
**Panel A: Shapes of the lumen at the beginning (green) of the expansion and at the maximum inflation pressure of 1.0 MPa for three different axial locations in the stent (shown in the middle left side).** Panel B: The corresponding von Mises contour maps.

**Figure 4:**
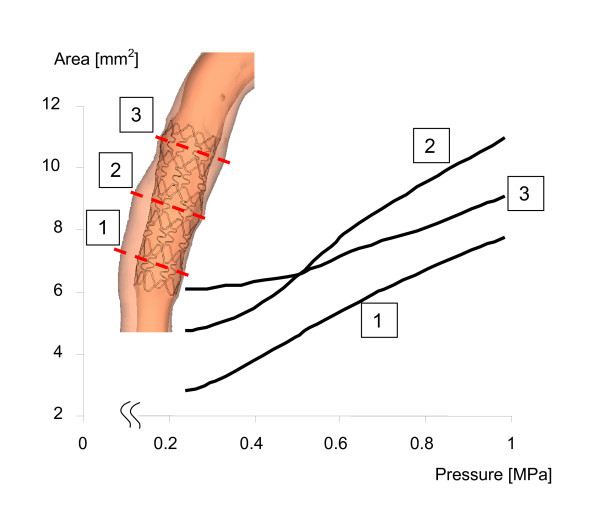
Expansion of the lumen area versus the inflation pressure for the three different axial locations in the stent depicted by the dotted red lines.

Figure 3 shows the results of the deployment procedure in three different locations at inflation pressures of 1.0 MPa. Stent deployment induced a hexagonal shaped lumen. The two proximal locations have a larger lumen area after stent deployment than the pre-intervention minimum luminal diameter (MLD) location. We can quantify this observation by looking at the pressure-diameter curves for these locations (Figure 4). The two most proximal location have lumen areas of 9.1 and 11.0 mm^2^, while lumen area at MLD is 7.8 mm^2^.

**Figure 5:**
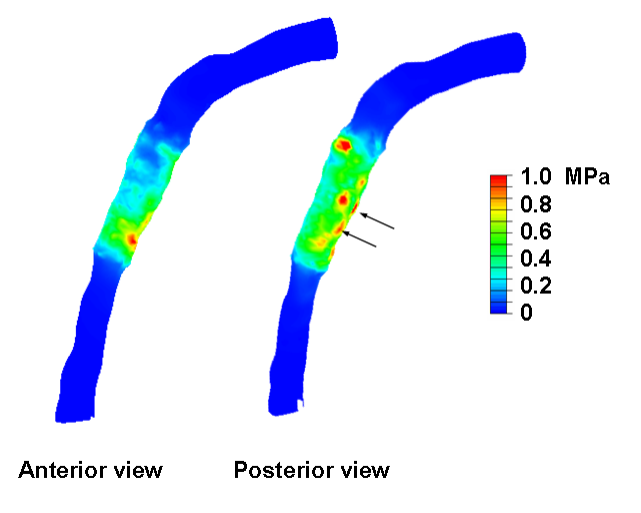
Von Mises contour maps in the anterior and posterior views of the luminal surface at the maximum inflation pressure of 1.0 MPa.

The stresses in the vessel wall at the three locations at maximum deployment pressure are also given in Figure 3. Generally, stresses decrease when going from the lumen wall interface radially outwards. Furthermore, stresses in the wall are higher when wall thickness decreases. The stresses at the luminal surface at inflation pressures of 1.0 MPa are shown in Figure 5. Stress peaks can be observed behind the stent struts, as indicated by the arrows. Due to the tapering of the vessel, average stresses increase slightly when going from proximal to distal. Several stress peaks can be observed, and they show a scattered pattern. Stress variations due to these scattered peaks are larger than the stress variations induced by tapering of the vessel.

**Figure 6:**
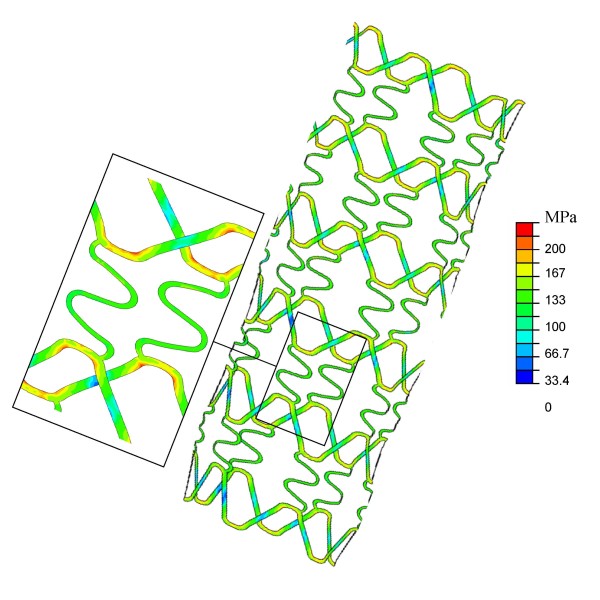
Von Mises stresses in the stent at the inflation pressure of 1.0 MPa.

The stresses in the stent struts are shown in Figure 6. Generally, average stresses in the stent struts seem to be somewhat higher in the proximal part of the stent, due to the larger displacements. Locally, highest stresses are observed near the connectors between the stent struts. These are the parts that are subjected to plastic deformation. Peak stresses in the stent struts approximate 200 MPa.

**Figure 7:**
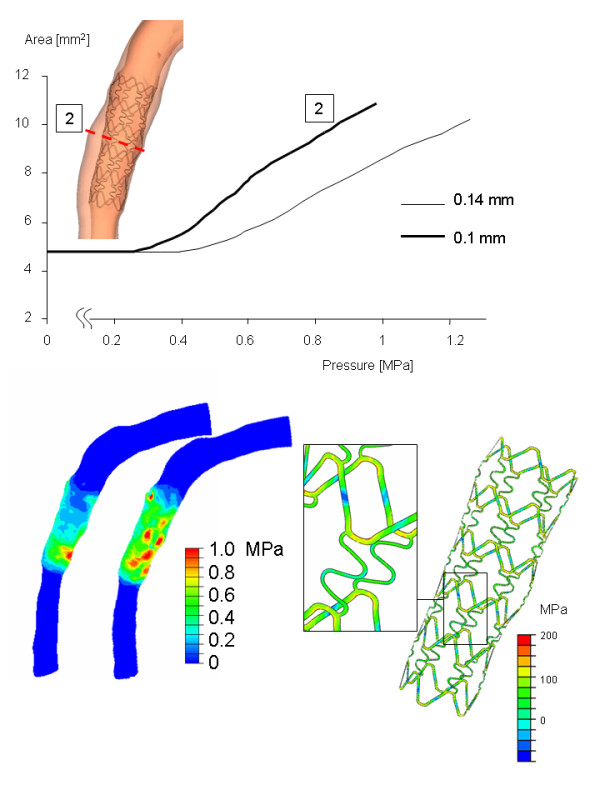
**Results from the simulation with a strut thickness of 0.14 mm.** The top panel shows the expansion versus the inflation pressure location 2. The panels at the bottom show the von Mises stress distribution at the luminal surface (left) and the stresses von Mises stress in the stent (right).

The results for the thicker stent struts are summarized in Figure 7. The increase in the strut thickness from 0.10 mm to 0.14 mm increases resistance of the stent to expansion. The pressure required to start the expansion of the stent increases. For the thicker stent struts, the central part of the stent will deform only if pressure is increased from 0.25 to 0.40 MPa. Furthermore, stent expansion will always be lower for the thicker stent struts for a given pressure. To reach the final expansion, inflation pressure had to be increased from 1.0 to 1.25 MPa. The stresses at the luminal surface are slightly elevated and the stress distribution is more heterogeneous. The combination of increased inflation pressure and thicker struts resulted in stresses which are comparable to the stresses in the thinner struts.

## Discussion

Computational structural analyses have emerged in the recent years as a tool to study the design of endovascular stents. Simulations of the interaction of stents and the arterial wall have also gained importance in predicting and calculating the stress state generated after a percutaneous intervention. This study is inserted in this field and illustrates the feasibility to apply numerical technologies to a patient-specific clinical scenario.

From the comparison with the preoperative situation it is possible to observe how the stent pushes the arterial wall towards the outside allowing the expansion of the occluded artery. The simulations also showed that the stresses are concentrated where the thickness of the arterial wall is minimal and behind stent struts. The stress peaks behind the stent struts coincide well with locations where increased neointimal hyperplasia was observed [2]. In the artery we used in this simulation, tapering influenced stress in the wall only minimally. The location with MLD is less expanded than the other locations in the stent: this can be expected since at MLD wall thickness is most likely larger than at other locations, and the presence of a thicker vessel wall can inhibit full stent deployment. Information like this could be useful to the interventional operator for the choice of inflation pressure and appropriate stent length.

From the computed deformation of the stent and the vessel wall one can observe larger deformation in the proximal part of the stent with maximum stress of 200 MPa, which is just marginally below the estimated fatigue fracture stress of the stent of 208 MPa [31]. Broken stent struts will increase vessel wall injury and could lead to in-stent restenosis, even in drug-eluting stents. Furthermore, these local injuries could induce stresses in the vessel wall that are large enough to perforate the vessel.

The results of the simulations also showed that thicker stent struts require higher inflation pressure, which might result in more damage to the vessel wall. Thinner struts are therefore preferable, especially since the stresses in the struts do not seem to be influenced significantly by the variation in strut thickness.

This study can surely be improved as it suffers from some limitations. From a modeling point of view the main are related to the absence of the angioplasty balloon and to the homogeneity of the arterial wall.

The absence of the balloon has an influence during the inflating process. Indeed, when the balloon is missing in a computational model, i.e. free expansion simulation, in absence of arterial interaction between stent and arterial wall the boundary conditions (load control, displacement control, presence of an inflating balloon) produce different results in terms of stent deformation, especially for the so call "dog-boning" effect [15]. Using load control as a boundary condition also prevents a direct comparison between inflation pressure from this study and clinically applied inflation pressure. However, the aim of this work was to show a computational approach to simulate the implantation of coronary stents in a realistic stenotic artery. When the artery is modeled we believe that the deformation of the stent at the end of the expansion phase produce results with smaller differences related to the boundary conditions applied. Furthermore, the stress values could slightly alter if more refined meshes are utilized. Future research will be devoted to remove this assumption to have a more realistic description of the stent deployment process.

Stresses in the arterial wall are related to arterial composition and injury. The heterogeneous material properties of the vessel wall should be inserted to better predict the deployment of a stent and the stresses in the vessel wall. However, the IVUS technique used in this study does not allow for discrimination between various wall components. Future application of IVUS-based virtual histology might be able to provide data on wall composition, which can then be included in the simulations. Combined with the appropriate material model for the components, the choice of which is still under debate due to lack of experimental data, a more accurate prediction of the wall stress distribution might be obtained. Besides the inhomogeneity of the wall, modeling of the plaque rupture mechanism should also receive attention. Indeed, fracture and/or micro-damage as well as damage to the arterial wall during stent deployment will change the mechanical properties of the vascular wall in terms of loss of stiffness. To our best knowledge, only three previous studies incorporated rupture mechanisms [13,32,33] but these models were based on a rough estimation of the tissue resistance to fissuring from available published and unpublished data. The inclusion of a fracture mechanism is beyond the purposes of this work, but should be addressed in future studies.

## Conclusion

In this study, we illustrated the feasibility to model stent deployment in a patient derived model of an atherosclerotic artery. Deployment of the stent was simulated by means of finite element method and clinically relevant inflation pressures were used. The current development of multislice computer tomography will allow non-invasive imaging for coronary arteries in the near future. Combined with these computer models, we will be able to simulate the outcome of a stenting procedure and select the appropriate stent to be implanted.

## Competing interests

The authors declare that they have no competing interests.

## Authors' contributions

FJHG and FM conceived the study, participated in the design of the study and drafted the manuscript. SS, LS and LP reconstructed the 3D stent model, built the finite element models and carried out the numerical simulations. AT, JW, AvdS, and PS contributed to the 3D reconstruction of the artery and JW critically revised the manuscript. GD participated in the design and coordination of the work and helped to revising critically the manuscript. All authors read and approved the final manuscript.
